# Intelligence

**Published:** 1821-12-01

**Authors:** 


### INTELLIGENCE.

The London Medical and Physical Journal is now conducted by Dr. A. B. Granville—*vice*, Dr. W. Hutchinson.

The London Medical Repository is henceforth to be conducted by Dr. Copland—*vice*, Drs. Uwins, Palmer, and Gray.

We are sorry that we have been forced to postpone, till our next number, the important reviews of Sir Astley Cooper's, and Mr. Wilson's Lectures—James on Inflammation—last volume of the Medico-Chirurgical Transactions—Transactions of the Physico-Medical Society of New York—Stevenson on Gutta Serena—Reid on Hypochondriasis—Chatelet and Martinet on Arachnitis—and the interesting review of the new Italian doctrines and practices. The above shall have precedency in our next number.

